# Preclinical Safety Evaluation of Intraperitoneally Administered Cu-Conjugated Anti-EGFR Antibody NCAB001 for the Early Diagnosis of Pancreatic Cancer Using PET

**DOI:** 10.3390/pharmaceutics14091928

**Published:** 2022-09-12

**Authors:** Hiroki Matsumoto, Chika Igarashi, Tomoko Tachibana, Fukiko Hihara, Mitsuhiro Shinada, Atsuo Waki, Sei Yoshida, Kenichiro Naito, Hiroaki Kurihara, Makoto Ueno, Kimiteru Ito, Tatsuya Higashi, Yukie Yoshii

**Affiliations:** 1 Institute for Quantum Medical Science, National Institutes for Quantum Science and Technology, Chiba 263-8555, Japan; 2 Department of Diagnostic Radiology, Kanagawa Cancer Center, Yokohama 241-8515, Japan; 3 Department of Chemistry, Graduate School of Science, Toho University, Chiba 274-8510, Japan; 4 Department of Research, NanoCarrier Co., Ltd., Tokyo 104-0031, Japan; 5 Department of Gastroenterology, Kanagawa Cancer Center, Yokohama 241-8515, Japan; 6 Department of Diagnostic Radiology, National Cancer Center Hospital, Tokyo 104-0045, Japan

**Keywords:** ^64^Cu-NCAB001, ipPET, preclinical safety, extended single dose toxicity study

## Abstract

Detecting tumor lesions <1 cm in size using current imaging methods remains a clinical challenge, especially in pancreatic cancer. Previously, we developed a method to identify pancreatic tumor lesions ≥3 mm using positron emission tomography (PET) with an intraperitoneally administered ^64^Cu-labeled anti-epidermal growth factor receptor (EGFR) antibody (^64^Cu-NCAB001 ipPET). Here, we conducted an extended single-dose toxicity study of ^64^Cu-NCAB001 ipPET in mice based on approach 1 of the current ICH M3 [R2] guideline, as our new drug formulation contains 45 μg of the antibody. We used NCAB001 labeled with stable copper isotope instead of ^64^Cu. The total content of size variants was approximately 6.0% throughout the study. The relative binding potency of Cu-NCAB001 to recombinant human EGFR was comparable to that of cetuximab. The general and neurological toxicities of Cu-NCAB001 ipPET at 62.5 or 625 μg/kg were assessed in mice. The no-observed-adverse-effect level of Cu-NCAB001 was 625 μg/kg, a dose approximately 1000-fold higher at the μg/kg level than the dose of ^64^Cu-NCAB001 in our formulation (45 µg). The size variants did not affect the safety of the formulation. Therefore, clinical studies on the efficacy of ^64^Cu-NCAB001 ipPET for early detection of pancreatic cancer using PET imaging can be safely conducted.

## 1. Introduction

Early detection and treatment of pancreatic cancer are major clinical challenges, considering that the overall 5-year survival rate of patients with pancreatic cancer is less than 10% [1,2,3,4]. To improve the patient survival rate, early detection and resection of tumor lesions <1 cm in size are critical; however, the efficacy of conventional imaging modalities in identifying small resectable pancreatic cancer is limited [5,6,7]. The epidermal growth factor receptor (EGFR) is a suitable target for the early diagnosis of pancreatic cancer with radiolabeled drugs and positron emission tomography (PET), because it plays a key role in regulating cell proliferation and is overexpressed in up to 90% of patients with pancreatic cancer [8,9]. Furthermore, intraoperative multi-instrument fluorescence imaging with IRDye800-conjugated anti-EGFR antibody cetuximab could be used to detect pancreatic cancer with a sensitivity of 96.1% [10]. Among the radionuclides used in PET imaging, we selected copper-64 (^64^Cu) to label EGFR because its half-life is suitable for evaluating the pharmacokinetics of radiolabeled antibodies in humans. In addition, it can be manufactured cost-effectively using a biomedical cyclotron [11]. In our preliminary studies, we labeled the cetuximab with ^64^Cu as it is clinically available. In an orthotopic xenograft mouse model of small resectable (<1 cm) pancreatic cancer, tumor lesions ≥ 3 mm were clearly identified using PET imaging through intraperitoneal (i.p.) administration of ^64^Cu-labeled cetuximab (^64^Cu-cetuximab ipPET) [12,13]. In contrast, intravenous (i.v.) administration of ^64^Cu-cetuximab or i.v./i.p. administration of ^18^F-fluorodeoxyglucose (^18^F-FDG) did not allow the detection of lesions of diameter ≥ 3 mm. To promote the use of this promising imaging procedure into clinical trials, we developed a new anti-EGFR antibody, NCAB001, in which the amino acid sequence is the same as that of cetuximab but the N-glycan profile is different (Supplementary Table S1 of [14]), and demonstrated that ^64^Cu-labeled NCAB001 (^64^Cu-NCAB001) is radiochemically and biochemically comparable to ^64^Cu-cetuximab [14]. Additionally, i.p. administration of ^64^Cu-NCAB001 to cynomolgus monkeys (*Macaca fascicularis*) was safely performed using ultrasound imaging [14]. We also assessed the biodistribution, radiation dosimetry, and pharmacologic profile of intraperitoneally administered ^64^Cu-NCAB001 (^64^Cu-NCAB001 ipPET) in non-human primates and found that it is well tolerated [14].

As our investigational new drug formulation ^64^Cu-NCAB001 contains 45 μg of NCAB001 [15], approach 1 of the current ICH M3 [R2] guidelines [16] was adopted for safety evaluation. An extended single-dose toxicity study in rodents using the same route as that intended for human administration is recommended when the total clinical dose used is less than 100 μg. We conducted such a study to assess the safety of intraperitoneally administered Cu-NCAB001, which is labeled with stable copper isotope instead of ^64^Cu in mice. In this study, in addition to the standard extended single-dose toxicity study with a recovery period of 14 days, we used Irwin’s test [17,18] to examine the potential effect of the test substance on the central nervous system, as recommended by the guidelines.

Here, we characterized and assessed the stability of Cu-NCAB001 throughout the toxicity study. More precisely, we investigated the presence of larger variants in the Cu-NCAB001 formulation and its binding potential to the target molecules. We here report the results of the abovementioned toxicity study. These results, together with our previous findings, could be used to assess the safety of ^64^Cu-NCAB001 ipPET, providing a basis for future PET clinical studies.

## 2. Materials and Methods

### 2.1. Ethics Statement

The experiments were performed in accordance with the laws and guidelines for animal welfare enacted by the Japanese Government and the National Research Council. All animal experiments were approved by the Institutional Animal Ethics Committee (approval code: G210284, approval date: 22 October 2021) and conducted in accordance with institutional guidelines. The expanded single-dose toxicity study complied with the good laboratory practice regulations.

### 2.2. Preparation, Characterization, and Stability of the Test Substance Cu-NCAB001

The anti-EGFR antibody NCAB001 was prepared according to the current good manufacturing practices (cGMP) at Mycenax Biotech Inc. (Jhunan, Taiwan), as previously reported [14]. For antibody conjugation, an NCAB001 solution (2 mg/mL in 50 mM borate buffer, pH 8.5) was prepared via buffer exchange with a Vivaspin ultrafiltration device (Sartorius, Göttingen, Germany). The bifunctional chelator 3,6,9,15-tetraazabicyclo[9.3.1]pentade-ca-1(15),11,13-triene-4-S-(4-isothiocyanatobenzyl)-3,6,9-triacetic acid (p-SCN-Bn-PCTA, Macrocyclics, Plano, TX, USA) was dissolved in dimethyl sulfoxide and added to the NCAB001 solution at a chelator-to-antibody molar ratio of 5:1. The mixtures were incubated overnight at 37 °C. After conjugation, the buffer was exchanged with PCTA-NCAB001 via ultrafiltration (PeliconXL Cassette Biomax 100 kDa, Merck Millipore, Burlington, MA, USA) with 0.1 M acetate buffer (pH 6.0) containing 100 mM glycine and 76.3 μM polysorbate-80, and the concentration of PCTA-NCAB001 was adjusted to 2 mg/mL. For Cu chelation with PCTA-NCAB001, CuCl_2_ dissolved in acetate buffer (0.1 M, pH 6.0) was added to the PCTA-NCAB001 solution at a ratio of 3:1 (*vol*:*vol*) and incubated for 1 h at 40 °C. Unbound Cu was removed via ultrafiltration.

The number of Cu-PCTA molecules conjugated to the antibody was determined using liquid chromatography–mass spectrometry (LC/MS), as previously reported [15]. The concentration of Cu in the Cu-NCAB001 solution was determined using inductively coupled plasma mass spectrometry (ICP-MS). Briefly, microwave pyrolysis of Cu-NCAB001 dissolved in nitric acid was conducted (ETHIOS-1, Milestone General, Kawasaki, Japan). This solution was dissolved in purified water and analyzed using ICP-MS (ELAN DRCII, Perkin Elmer, Waltham, MA, USA). Size variants in NCAB001 and Cu-NCAB001 solutions were determined using size-exclusion high-performance liquid chromatography (SEC-HPLC). An LC system (LC-30A, Shimadzu Corporation, Kyoto, Japan) and a TSKgel G3000 SWXL column (300 mm × 7.8 mm, 5 μm, Tosoh Bioscience, Tokyo, Japan) were used. Elution was conducted using potassium phosphate buffer containing 250 mM potassium chloride at a flow rate of 0.5 mL/min and was monitored at a UV wavelength of 280 nm. The relative binding potency of Cu-NCAB001 to recombinant human EGFR (R&D Systems, Minneapolis, MN, USA) was determined using enzyme-linked immunosorbent assay (ELISA) with cetuximab (Merck Biopharma, Tokyo, Japan) as a reference standard, as previously reported [15].

SEC-HPLC and ELISA were performed before and after the toxicity study. The results were compared, and the stability of the test substance Cu-NCAB001 throughout the toxicity study was confirmed.

### 2.3. Animals and Housing

Five-week-old male and female ICR mice were obtained from Charles River Laboratories Japan (Atsugi, Japan). The mice were housed individually in polycarbonate bins containing appropriate bedding. The room temperature was maintained at 23 ± 3 °C with a relative humidity of 50% ± 20%, and a 12 h light/dark cycle. Solid food (Oriental Yeast Co. Ltd., Tokyo, Japan) and municipal tap water were provided to the mice ad libitum. The mice were weighed and randomized into experimental groups after 7 days of quarantine. No known contaminants that could interfere with the results of the study existed in the administered food or water.

### 2.4. Single-Dose Extended Toxicity Study of Intraperitoneally Administered Cu-NCAB001 in Mice

In total, 40 male and 40 female mice were randomized into single-sex groups based on their body weight. A single dose of the following substances was administered by i.p. injection to the experimental groups: vehicle (n = 15 of each sex), 62.5 μg/kg Cu-NCAB001 (n = 10 of each sex), or 625 μg/kg Cu-NCAB001 (n = 15 of each sex). A solution of acetate buffer 0.1 M (pH 6.0), containing 100 mM glycine and 76.3 μM polysorbate-80, was used as the vehicle. These doses are expected to provide appropriate systemic exposure multiples of doses intended for clinical use. The doses 62.5 and 625 μg/kg were considered to be approximately 100- and 1000-fold higher than the clinical doses, respectively, on a μg/kg basis.

The schedule of the study assessments is summarized in Table 1. On day 0, cage-side observations were performed pre-dosing, and 5 min, 15 min, 30 min, 1 h, 2 h, 4 h, and 8 h after the administration of the substances. The cage-side observations were repeated once a day before noon on day 1 and thereafter. Body weight and food consumption were evaluated before noon on days 1, 3, 7, 10, and 14. Neurological observations were performed according to Irwin’s test [17,18] on three separate days (before the administration, 30 min after the administration on day 0, and 24 h after the administration on day 1), using five animals of each sex and group. Urine samples were collected for urinalysis on day 11, and blood samples were collected on the designated day of sacrifice for each group for hematological assessment and serum biochemistry. Urinalysis was performed using an automatic urine analyzer (CLINITEK 500; Siemens Healthineers, Erlangen, Germany). Hematological parameters were measured using a hematological analyzer (ADVIA 2120i; Siemens Healthineers, Erlangen, Germany). Serum biochemical parameters were measured using a blood biochemical analyzer (TBA 120FR; Canon Medical Systems, Tokyo, Japan). The mice were sacrificed on the designated day for each group and necropsy was performed. The brain, thymus, heart, lungs, liver, kidneys, and testes were excised and weighed. Histopathological evaluations of the cerebrum, cerebellum, heart, lung, stomach, duodenum, jejunum, ileum, cecum, colon, rectum, liver, kidney, testis/ovary, femur, and macroscopically abnormal sites were performed. As there were no significant differences in histopathological observations between the 625 μg/kg-treated and control groups on day 1, the 62.5 μg/kg-treated group was not subjected to all assessments of delayed toxicity and recovery from day 2 to day 14.

Specific items observed as a part of Irwin’s test are listed below (Table 2).

### 2.5. Statistical Analysis

Data are expressed as mean with the corresponding standard deviation (SD). Bartlett’s and Dunnett’s tests were performed for multiple group comparisons. Steel’s test was performed in case of heterogeneous group variances. For two-group comparisons, the F-test and Student’s *t*-test were performed. For heterogeneous group variances and unequal n’s, Welch’s *t*-test was performed. Results with *p* < 0.05 were considered statistically significant.

## 3. Results

### 3.1. Preparation, Characterization, and Stability of the Test Substance Cu-NCAB001

**Figure 1:**
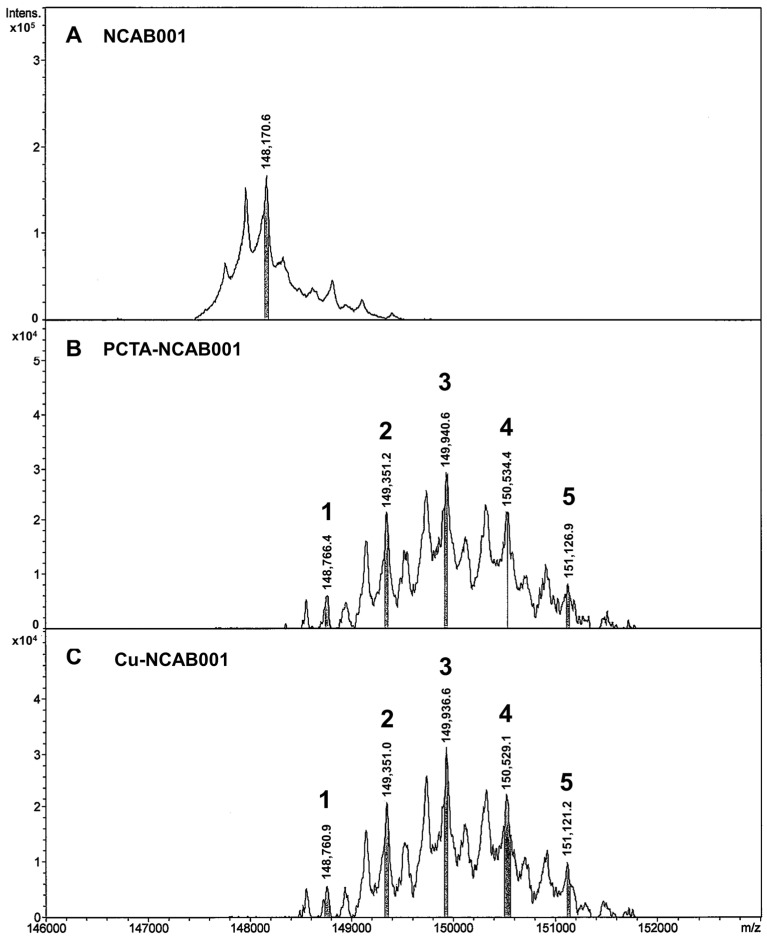
Deconvoluted mass spectra of NCAB001 (**A**), PCTA-NCAB001 (**B**), and Cu-NCAB001 (**C**). The numbers on the spectra denote the numbers of PCTA (**B**) or Cu-PCTA (**C**) molecules conjugated to NCAB001 (average of 3, range from 1 to 5).

**Figure 2:**
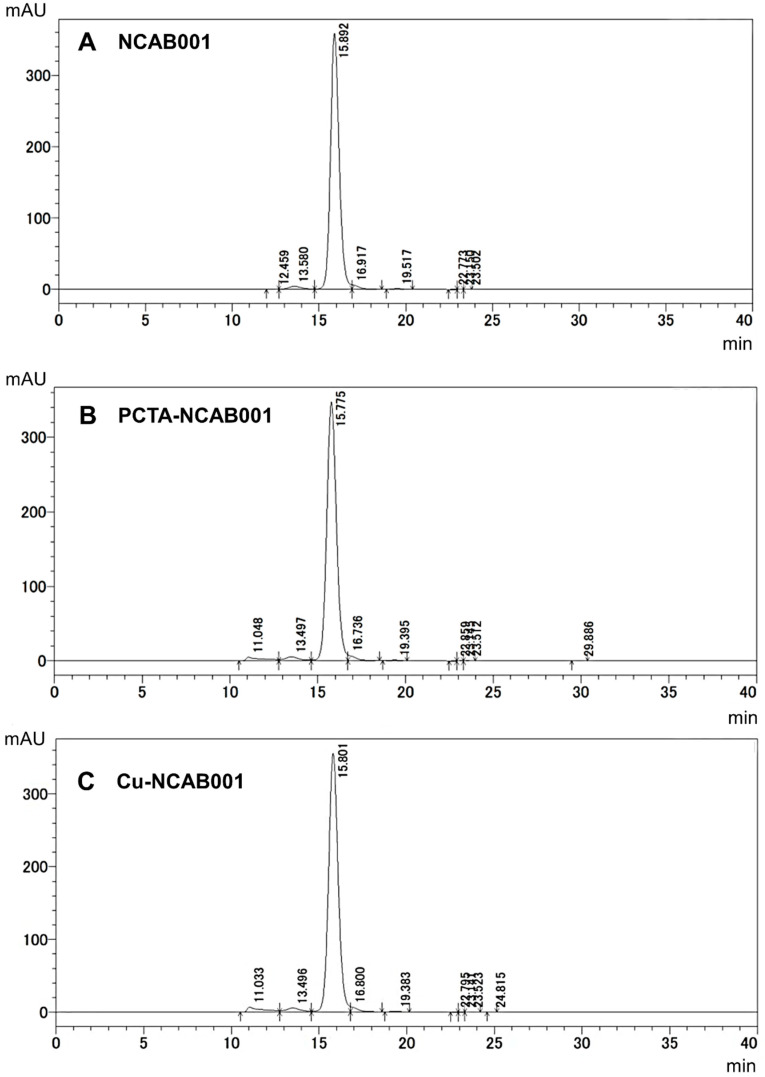
Size variants of NCAB001 (**A**), PCTA-NCAB001 (**B**), and Cu-NCAB001 (**C**). A larger impurity was found in NCAB001 solution at the retention time of 13 min. An additional larger impurity was found in the PCTA-NCAB001 and Cu-NCAB001 solutions at a retention time of 11 min.

**Figure 3:**
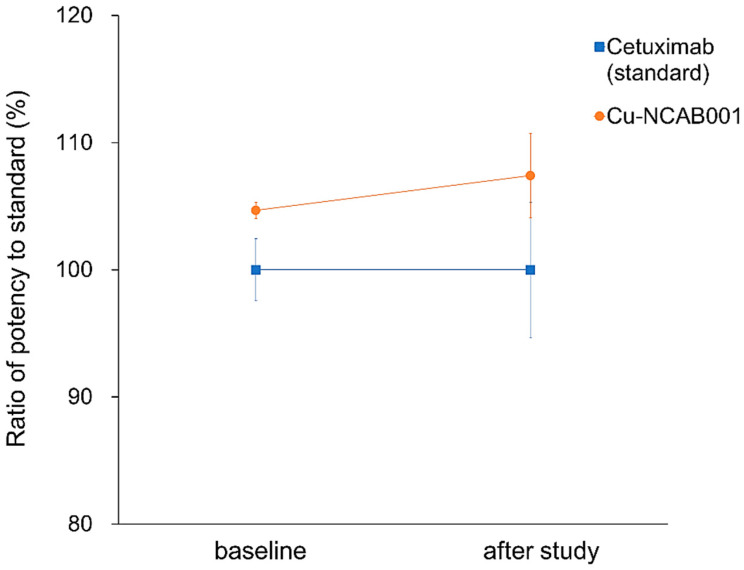
Relative binding potency of Cu-NCAB001 to recombinant human EGFR before and after the toxicity study was determined using the enzyme-linked immunosorbent assay (ELISA). Cetuximab was used as the reference standard. No statistically significant changes were observed.

The anti-EGFR antibody NCAB001 was manufactured according to cGMP regulations and was further conjugated to PCTA, followed by chelation with copper ions. The mass spectra of NCAB001, PCTA-NCAB001, and Cu-NCAB001 were obtained using LC/MS, and the number Cu-PCTA molecules conjugated to NCAB001 was calculated from the deconvoluted spectra. On an average, approximately three Cu-PCTA molecules (in the range of 1 to 5; Figure 1) were conjugated to NCAB001. The concentration of Cu in the 2 mg/mL Cu-NCAB001 solution was 3.2 μg/mL, as determined using ICP-MS. Thus, an average of 3.7 molar equivalents of Cu was chelated to the PCTA-NCAB001 conjugate. The size variants of NCAB001 and Cu-NCAB001 in the solutions were analyzed using SEC-HPLC (Figure 2). A larger variant comprised approximately 1% of the NCAB001 formulation (Figure 2A, retention time 13 min). Another variant of a larger size comprised approximately 2% of the PCTA-NCAB001 and Cu-NCAB001 formulation (Figure 2B,C, retention time 11 min). Before the extended single-dose toxicity study, the Cu-NCAB001 solution contained up to 6.0% total size variants. Additional SEC-HPLC was conducted after the toxicity study, and the percentage of total size variants in the solution was 5.7%. The relative binding potency of Cu-NCAB001 to recombinant human EGFR was determined using ELISA. The potency of Cu-NCAB001 was comparable to that of cetuximab, which was used as the reference standard, before and after the toxicity study (Figure 3). The results of SEC-HPLC and ELISA showed that the quality of the test substance Cu-NCAB001 was maintained throughout the toxicity study. The specifications of the Cu-NCAB001 solution are summarized in Table 3.

### 3.2. Single-Dose Extended Toxicity Study of Intraperitoneally Administered Cu-NCAB001 in Mice

#### 3.2.1. In-Life Observations

**Figure 4:**
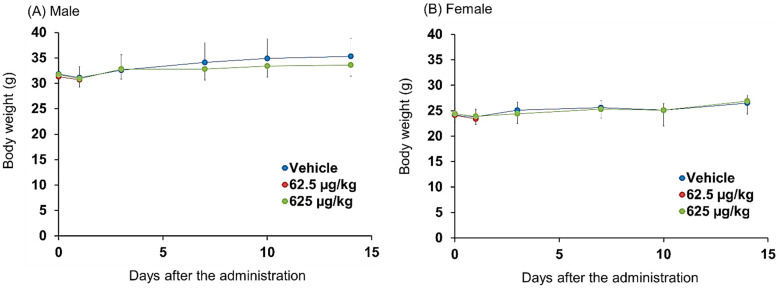
Changes in the body weights of mice after the intraperitoneal administration of Cu-NCAB001. Plots of the average body weight of (**A**) male and (**B**) female mice in each treatment group of the extended single-dose toxicity study are shown. Values are presented as mean ± SD. The means were calculated based on the following number of values: days 0 and 1, vehicle and 625 µg/kg groups, *n* = 15; 62.5 µg/kg group, *n* = 10; day 3 and thereafter, vehicle and 625 µg/kg groups, *n* = 5. As there were no significant differences in histopathological observations between the 625 μg/kg-treated and control groups on day 1, the 62.5 μg/kg-treated group was not subjected to the assessment of body weight from day 2 to day 14. No statistically significant changes were observed.

**Figure 5:**
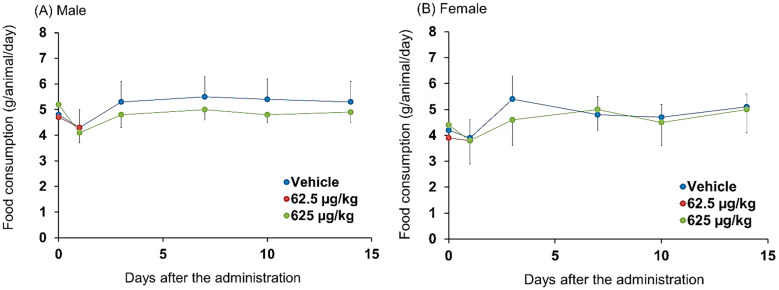
Changes in food consumption of mice after the intraperitoneal administration of Cu-NCAB001. (**A**) Male. (**B**) Female. Plots of average food consumption for each treatment group in the extended single-dose toxicity study are shown. Values are presented as mean ± SD. The means were calculated based on the following number of values: days 0 and 1, vehicle and 625 µg/kg groups, n = 15; 62.5 µg/kg group, n = 10; day 3 and thereafter, vehicle and 625 µg/kg groups, n = 5. As there were no significant differences in histopathological observations between the 625 μg/kg-treated and control groups on day 1, the 62.5 μg/kg-treated group was not subjected to assessment of food consumption from day 2 to day 14. No statistically significant changes were observed.

No treatment-related signs of toxicity were observed. There were no significant differences in body weight (Figure 4A,B) or food consumption (Figure 5A,B) between the treated and control groups. Neurological observations were performed according to Irwin’s test (Table 2).

#### 3.2.2. Pathology Assessment (Urinalysis, Hematology, and Serum Biochemistry)

Table 4 summarizes the urinalysis results. No treatment-related changes were observed in the treated groups compared with the control.

**Figure 6:**
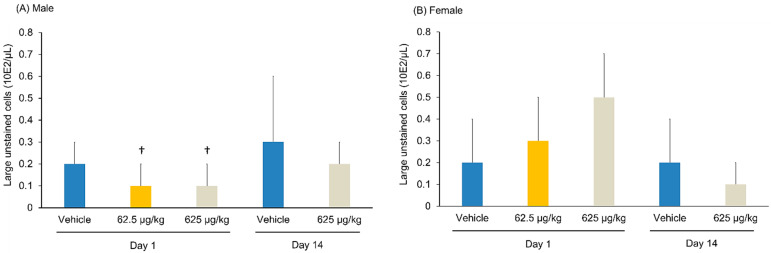
Changes in the number of large unstained cells in the blood after the intraperitoneal administration of Cu-NCAB001 into mice. (**A**) Male. (**B**) Female. Values are presented as mean ± SD. The means were calculated based on the following number of values: day 1, vehicle and 625 µg/kg groups, n = 15; 62.5 µg/kg group, n = 10; day 14, vehicle and 625 µg/kg groups, n = 5. As there were no significant differences in histopathological observations between the 625 μg/kg-treated and control groups on Day 1, the 62.5 μg/kg-treated group was not subjected to assessment on Day 14. ^†^  *p* ≤ 0.05 vs. vehicle group on the same day (Dunnett test, two-side).

There were no significant changes in hematological parameters between the treated and control groups. On day 1, a significant decrease in the number of large unstained cells was observed in the male mice of the 62.5 and 625 μg/kg-treated groups compared with that in the control mice (Figure 6). However, the individual results were within the normal accepted limits. Therefore, this finding was not considered relevant. The hematological parameters of each group are summarized in Supplementary Tables S1-1 and S1-2.

**Figure 7:**
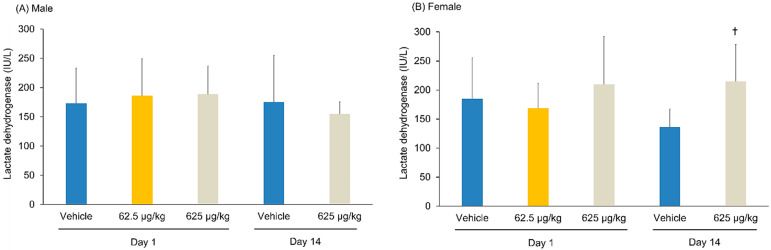
Changes in the activity of lactate dehydrogenase in the blood after the intraperitoneal administration of Cu-NCAB001 into mice. (**A**) Male. (**B**) Female. Values are presented as mean ± SD. The means were calculated based on the following number of values: day 1, vehicle and 625 µg/kg groups, n = 15; 62.5 µg/kg group, n = 10; day 14, vehicle and 625 µg/kg groups, n = 5. As there were no significant differences in histopathological observations between the 625 μg/kg-treated and control groups on day 1, the 62.5 μg/kg-treated group was not subjected to assessment on day 14. ^†^ *p* ≤ 0.05 vs. vehicle group on the same day (*t*-test, two-side).

There were no significant changes in the serum biochemical parameters between the treated and control groups. On day 14, a significant increase in the lactate dehydrogenase level was observed in the female mice of the 625 μg/kg group compared with that in the control mice (Figure 7). However, the individual results were within the normal accepted limits. Therefore, this finding was not considered relevant. The serum biochemical parameters of each group are summarized in Supplementary Tables S2-1 and S2-2.

#### 3.2.3. Organ Weights, Necropsy, and Histopathological Observations

There were no significant changes in the organ weights between the treated and control groups. On day 1, a significant increase in the kidney weight was observed in the female mice of the 625 μg/kg group compared with that in the control. However, the individual results were within the normal accepted limits. Therefore, this finding was not considered relevant. The organ weights in each group are summarized in Supplementary Tables S3-1 and S3-2. There were no observable treatment-related changes between the organs of the treated animals and those of the control, according to necropsy.

Histopathological examinations were conducted for the group treated with the highest dose of the test substance (625 μg/kg) and the control group. There were no treatment-related histopathological changes in the 625 μg/kg-treated group compared with those in the control group. No adverse effects were observed at the administration site in either group. As there were no significant differences in histopathological observations between the 625 μg/kg-treated and control groups, the 62.5 μg/kg-treated group was not subjected to histopathological assessment. The histopathological findings for the two evaluated groups are summarized in Table 5.

## 4. Discussion

In this study, we evaluated the preclinical safety of intraperitoneally administered Cu-NCAB001 using an expanded single-dose toxicity study in mice. We also characterized the test substance Cu-NCAB001 and assessed its stability throughout the toxicity study.

In our previous studies, we demonstrated that the i.p. administration of ^64^Cu-cetuximab allows the detection of the early stages of pancreatic cancer in mouse models using PET. In these studies, we used clinically available cetuximab (Erbitux) as the starting material for this radiopharmaceutical [11,12,19]. To promote the use of this approach in clinical practice, we developed a new anti-EGFR antibody, NCAB001, which possesses the same amino acid sequence as cetuximab, but a different N-glycan profile (Supplementary Table S1 of [14]). We previously demonstrated that ^64^Cu-NCAB001 is radiochemically and biochemically comparable to ^64^Cu-cetuximab [13]. Additionally, ^64^Cu-NCAB001 was safely administered by i.p. injection to monkeys using ultrasound imaging [13]. In that study, each dose of ^64^Cu-NCAB001 contained 100 μg of NCAB001, which was approximately 7000-fold less than the single dose of cetuximab (400 mg/m^2^, i.v. drip infusion) used in clinical practice [20]. The cardiovascular parameters were within the reference ranges before and after the i.p. administration of ^64^Cu-NCAB001, throughout the observation period. Administration-related changes suggesting local irritation or toxicity, such as intestinal inflammation, were not observed at the injection site or in the organs located in the intraperitoneal cavity. PET images showed that ^64^Cu-NCAB001 was spread throughout the intraperitoneal cavity for 6 h following i.p. administration and was cleared thereafter. The internal dose was relatively high in the liver, 0.107 mSv/MBq; for other organs in the peritoneal cavity, it was 0.03–0.09 mSv/MBq. The estimated effective dose of 130 MBq ^64^Cu-NCAB001 ipPET is equivalent to the doses of other radiotracers, such as ^18^F-FDG PET, used in current clinical PET procedures [21].

Our investigational new drug formulation, ^64^Cu-NCAB001, contains 45 μg of NCAB001 [15]. Here, we conducted an extended single-dose toxicity study of ^64^Cu-NCAB001, after i.p. administration in mice, in accordance with the ICH M3(R2) guideline (Approach 1) [16]. On an average, three Cu-PCTA molecules corresponding to 3.7 molar equivalents of Cu, were conjugated to NCAB001. SEC-HPLC indicated the presence of one larger variant in the NCAB001 formulation. Another large variant was formed during the conjugation of Cu with NCAB001. The total content of size variants contained in the Cu-NCAB001 solution was approximately 6.0%, based on the relative peak area of the chromatogram, throughout the extended single-dose toxicity study. The relative binding potency of Cu-NCAB001 to recombinant human EGFR was stable throughout the toxicity study and comparable to that of cetuximab. The results of SEC-HPLC and ELISA showed that the quality of the test substance Cu-NCAB001 was maintained throughout the toxicity study.

Single doses of 62.5 or 625 μg/kg ^64^Cu-NCAB001 were administered intraperitoneally to mice, and their general toxicity was compared with that of the vehicle. No treatment-related changes in cage-side observations, body weights, food consumption, urinalysis, hematological and serum biochemical parameters, organ weights, or histopathological examinations were observed between the groups. A significant decrease in the number of large unstained cells was observed in the male mice of the 62.5 and 625 μg/kg-treated groups compared with that in the control mice on day 1 (Figure 6). This change was not observed in the male mice on day 14, nor in the female mice on day 1 and day 14. A significant increase in the lactate dehydrogenase level was observed in the female mice of 625 μg/kg group compared with that in the control mice on day 14 (Figure 7). However, this level was not changed in the female mice of 625 μg/kg group from day 1 to day 14. In addition, this change was not observed in the male mice. A significant increase in the kidney weight was observed in the female mice of the 625 μg/kg group compared with that in the control on day 1 (Supplementary Table S3-2). However, kidney weight was not changed in this group from day 1 to day 14, and the kidney weight relative to the whole-body weight was not changed in this group. In addition, individual results of these parameters were within the normal accepted limits. Therefore, these findings were not considered relevant to the administration of Cu-NCAB001. Following necropsy and during histopathological investigation, we carefully examined the administration site and organs located in the peritoneal cavity, and no adverse effects were observed in the treated group compared with the control. Based on these results, the no-observed-adverse-effect level of Cu-NCAB001 was 625 μg/kg. This dose is approximately 1000-fold higher at the μg/kg level than the doses of Cu-NCAB001 used in our current investigational drug formulation.

For diagnostic radiopharmaceuticals made of biological products, safety pharmacology studies should assess the presence of any functional effects on the cardiovascular, respiratory, and central nervous systems [22,23]. Previously, we evaluated the effect of ^64^Cu-NCAB001 administered intraperitoneally at a dose of 100 μg/2.5 kg body (i.e., 40 μg/kg) on the cardiovascular and respiratory systems, in monkeys [14]. In this study, in addition to the standard extended single-dose toxicity study, we evaluated the effect of Cu-NCAB001 intraperitoneally administered on the central nervous system at doses of 62.5 and 625 μg/kg. These doses were similar to those used in our previous study. The neurological observations listed in Table 2 were based on Irwin’s test [17,18]. There were no changes between the treatment and vehicle groups. It is also known that cetuximab can cause dermatologic toxicities such as acneiform rash [20]. In this study, we carefully observed the skin during necropsy, and treatment-related changes on the skin were not observed. Our previous study involving monkeys also indicated no observable treatment-related changes on the skin [14]. Therefore, it is suggested that the intraperitoneal administration of Cu-NCAB001 has relatively low risk on the skin. We also assessed the biodistribution of intraperitoneally administered ^64^Cu-NCAB001 in mice and monkeys, and estimated the radiation dosimetry in human [14]. The estimated effective dose is equivalent to those of the current clinical PET examinations. These results, together with our previous findings, will be used for the risk assessment of ^64^Cu-NCAB001, offering essential information for the elaboration of future PET clinical studies.

In this toxicity study, we used a formulation of the test substance containing approximately 5–6% of various size variants. These size variants are suggested to be a dimer or oligomers of the NCAB001 antibody, and the identification of these size variants are in progress. Our results suggest that their presence does not affect the safety of ^64^Cu-NCAB001. Process development studies are currently in progress to remove the size variants of PCTA-NCAB001. A total content of size variants below 5% will be used as specification for the future products.

This study had some limitations. The size variants of ^64^Cu-NCAB001 should have been identified. Additionally, the safety of the i.p. administration of ^64^Cu-NCAB001 with the aid of ultrasound imaging will have to be thoroughly assessed in our future clinical trials.

## 5. Conclusions

This extended single-dose toxicity study of Cu-NCAB001 intraperitoneally administered in mice demonstrated that the no-observed-adverse-effect level of the tested substance is 625 µg/kg. The formulation contained up to 6% different size variants; however, this did not affect its safety. These results and those of previous studies indicate clinical studies on the efficacy of ^64^Cu-ATSM intraperitoneally administered at a dose of 45 µg for early detection of pancreatic cancer by PET imaging can be safely conducted in the near future.

## Figures and Tables

**Table 1:** Schedule of endpoint collections and study timeline.

Days after Administration	−3	0	1	2	3	→	7	→	10	11	→	14
Administration		√										
Cage-side observations ^1^		√	√	†	†	†	†	†	†	†	†	†
Body weight		√	√		†		†		†			†
Food consumption		√	√		†		†		†			†
Irwin’s test ^2^	√	√	√									
Urinalysis										†		
Hematology, serum biochemistry			‡									†
Sacrifice ^3^			‡									†

√: All groups, n = 15 for each sex and group. ‡: All groups, n = 10 for each sex and group. † Vehicle and 625 µg/kg groups, n = 5 for each sex and group. →: Cage-side observations were made on day 4, 5, 6, 8, 9, 12, and 13. As there were no significant differences in histopathological observations between the 625 μg/kg-treated and control groups on day 1, the 62.5 μg/kg-treated group was not subjected to all assessments of delayed toxicity and recovery from day 2 to day 14. ^1^ General conditions such as external appearance, nutritional status, posture, behavior, and abnormalities in excrement production were observed before administration and 5 min, 15 min, 30 min, 1 h, 2 h, 4 h, and 6 h after administration on day 0, once a day in the morning on day 1 and thereafter. ^2^ Neurological conditions as listed in Table 2 were observed 3 days before the administration, 30 min after the administration on day 0, and 24 h after the administration on day 1. ^3^ Organ weights, necropsy, and histopathological observations.

**Table 2:** Irwin’s Test: Observations.

Category	Parameter	Category	Parameter
Awareness	Alertness	Muscle tone	Limb tone
	Visual placing		Body sag
	Stereotypy		Body tone
	Passivity	Reflexes	Pinna reflex
Mood	Grooming		Corneal reflex
	Vocalization		Ipsilateral flexor reflex
	Irritability	Autonomic profile	Writhing
	Restlessness		Pupil size
	Fearfulness		Palpebral size
Motor activity	Reactivity		Exophthalmos
	Spontaneous activity		Urination
	Touch response		Salivation
	Pain response		Piloerection
CNS excitation	Startle response		Body temperature
	Straub’s tail reaction		Skin color
	Tremor		Heart rate
	Twitches		Respiratory rate
	Convulsion		
Motor incoordination	Body position		
	Limb position		
	Staggering gait		
	Abnormal gait		
	Surface righting reflex		

**Table 3:** Specifications of the Cu-NCAB001 solution.

Type	Test	Method	Result
Protein concentration		UV absorption at 280 nm	2.28 mg/mL
pH		Japanese Pharmacopeia	6.05
Potency	Antigen-binding activity	ELISA	105 ± 0.2% of Erbitux
Purity/impurity	Size variants	SEC-HPLC	Impurity: 6.7%
Confirmation	Molecular weight	TOF-MS	Average of three molecules of Cu-PCTA conjugated to NCAB001
	Cu concentration	ICP-MS	3.2 µg/mL
Buffer composition			
0.1 M acetate buffer (pH 6.0) containing 100 mM glycine and 76.3 μM polysorbate-80			

ELISA: enzyme immunosorbent assay; SEC-HPLC: size-exclusion high-performance liquid chromatography; TOF-MS: time-of-flight mass spectrometer; ICP-MS: inductively coupled plasma mass spectrometry.

**Table 4:** Results of urinalysis (day 11 after administration).

Sex	Male		Female	
Dose (µg/kg)	0	625	0	625
No. of Animals	5	5	5	5
pH				
≥7.0	0	0	0	0
7.5	0	1	0	1
8.0	1	1	1	1
8.5	4	3	3	3
≤9.0	0	0	1	0
Proteins				
(−)	0	0	1	3
(±)	2	4	4	2
(1+)	3	1	0	0
(2+)	0	0	0	0
Ketones				
(−)	0	0	2	3
(±)	2	4	3	2
(1+)	3	1	0	0
(2+)	0	0	0	0
Glucose				
(−)	5	5	5	5
(1+)	0	0	0	0
(2+)	0	0	0	0
Occult blood				
(−)	5	5	4	4
(±)	0	0	0	0
(1+)	0	0	1	1
(2+)	0	0	0	0
Urobilinogen				
(±)	5	5	5	5
(1+)	0	0	0	0
Color				
Light yellow	0	0	0	0
Yellow	5	5	5	5
Dark yellow	0	0	0	0
Brown	0	0	0	0

Protein) −: Negative, ±: 15, 1+: 30, 2+: ≥100 mg/dL. Ketones) −: Negative, ±: 5, 1+: 15, 2+: ≥40 mg/dL. Glucose) −: Negative, 1+: 100, 2+: ≥250 mg/dL. Occult blood) −: Negative, ±: 0.015, 1+: 0.062. 2+: ≥0.135 mg/dL. Urobilinogen) ±: 0.1–1.0, 1+: ≥2.0 Ehrlich U/dL.

**Table 5:** Histopathological findings.

Days after Dosing	1				14			
Sex	Male		Female		Male		Female	
Dose (µg/kg)	0	625	0	625	0	625	0	625
No. of animals	10	10	10	10	5	5	5	5
Gross Pathological Findings								
All tissues								
Not remarkable	10	10	10	10	5	5	5	5
Heart								
Infiltrate, inflammatory cell	0	0	1	0	0	0	0	0
Minimal	0	0	1	0	0	0	0	0
Kidney								
Cyst	0	1	0	0	0	0	0	0
Minimal	0	1	0	0	0	0	0	0
Basophilia, tubule	1	0	0	1	0	0	0	0
Minimal	1	0	0	1	0	0	0	0
Testis								
Atrophy, tubule	1	0	N/A	N/A	0	0	N/A	N/A
Mild	1	0			0	0		

The organs not listed in this table did not have any remarkable microscopic findings. N/A: Not applicable.

## Data Availability

All data are presented within the article and the Supplementary Materials.

## Supplementary Materials

The following supporting information can be downloaded at https://www.mdpi.com/article/10.3390/pharmaceutics14091928/s1, Supplementary Table S1: Hematological parameters after intraperitoneal administration of Cu-NCAB001 in mice; Table S2: Serum biochemical parameters after intraperitoneal administration of Cu-NCAB001 in mice; Table S3: Organ weights after intraperitoneal administration of Cu-NCAB001 in mice.
