# Supplementary material for: Preclinical Safety Evaluation of Intraperitoneally Administered Cu-Conjugated Anti-EGFR Antibody NCAB001 for the Early Diagnosis of Pancreatic Cancer Using PET
Source: Pharmaceutics. 2022 Sep 12;14(9):1928. doi: 10.3390/pharmaceutics14091928 (PMC9504034; doi:10.3390/pharmaceutics14091928)
Supplement: Supplementary file 1 [file pharmaceutics-14-01928-s001.zip › pharmaceutics-1858767-supplementary.pdf]

pharmaceutics

MDPI

Supplementary Materials

Preclinical Safety Evaluation of Intraperitoneally Administered Cu-Conjugated Anti-EGFR Antibody NCAB001 for the Early Diagnosis of Pancreatic Cancer using PET

Table S1-1. Hematological parameters after intraperitoneal administration of Cu-NCAB001 in male mice.

Parameters	Day 1 (n = 10)			Day 14 (n = 5)	
	Vehicle	62.5 μg/kg	625 μg/kg	Vehicle	625 μg/kg
RBC 10E4/μL	908 ± 33	899 ± 46	892 ± 37	925 ± 23	928 ± 55
HGB g/dL	14.6 ± 0.4	14.5 ± 0.6	14.3 ± 0.5	14.6 ± 0.2	14.8 ± 0.8
HCT %	48.3 ± 1.4	47.3 ± 1.9	47.0 ± 1.3	47.0 ± 0.5	47.9 ± 2.5
MCV fL	53.3 ± 1.6	52.7 ± 1.0	52.8 ± 1.7	50.9 ± 0.8	51.5 ± 1.4
MCH pg	16.1 ± 0.5	16.2 ± 0.4	16.0 ± 0.5	15.8 ± 0.2	15.9 ± 0.6
Reticulocyte %	3.3 ± 0.4	3.2 ± 0.5	3.3 ± 0.5	3.3 ± 0.6	2.8 ± 0.3
Reticulocyte 10E9/L	296.4 ± 36.2	286.1 ± 45.2	293.3 ± 44.8	307.9 ± 67.2	264.4 ± 33.0
PLT 10E4/μL	129.3 ± 31.0	130.6 ± 12.6	131.5 ± 13.7	124.4 ± 27.3	119.4 ± 10.1
WBC 10E2/μL	51.2 ± 17.0	38.2 ± 11.4	45.8 ± 12.0	34.6 ± 21.3	40.0 ± 2.8
LYMP %	78.3 ± 7.6	75.1 ± 6.6	74.7 ± 4.2	77.7 ± 4.2	74.0 ± 6.9
NEUT %	16.9 ± 6.4	20.0 ± 5.9	20.9 ± 4.3	20.0 ± 5.9	20.9 ± 4.3
EOS %	3.2 ± 1.6	3.4 ± 1.2	2.7 ± 1.0	2.6 ± 0.5	3.3 ± 1.1
BASO %	0.2 ± 0.1	0.1 ± 0.1	0.2 ± 0.1	0.1 ± 0.1	0.1 ± 0.0
MONO %	1.1 ± 0.4	1.0 ± 0.7	1.1 ± 0.5	1.4 ± 0.7	1.6 ± 0.5
LUC %	0.5 ± 0.2	0.4 ± 0.2	0.4 ± 0.1	0.7 ± 0.3	0.5 ± 0.2
LYMP 10E2/μL	40.6 ± 15.3	28.4 ± 8.1	34.5 ± 10.6	26.5 ± 15.3	29.5 ± 3.3
NEUT 10E2/μL	8.1 ± 2.6	7.8 ± 3.4	9.3 ± 1.9	6.3 ± 4.9	8.2 ± 2.6
EOS 10E2/μL	1.6 ± 1.0	1.3 ± 0.7	1.2 ± 0.4	0.9 ± 0.5	1.3 ± 0.4
BASO 10E2/μL	0.1 ± 0.1	0.0 ± 0.1	0.0 ± 0.1	0.0 ± 0.0	0.0 ± 0.0
MONO 10E2/μL	0.5 ± 0.2	0.4 ± 0.4	0.5 ± 0.3	0.5 ± 0.5	0.6 ± 0.2
LUC 10E2/μL	0.2 ± 0.1	0.1 ± 0.1†	0.1 ± 0.1†	0.3 ± 0.3	0.2 ± 0.1

Values are shown as the mean±SD. †$p \leq 0.05$ vs. vehicle group (Dunnett test, two-side). RBC: Red Blood Cells, HGB: Hemoglobin, HCT: Hematocrit, MCV: Mean Red Blood Cell Volume, MCH: Mean Red Blood Cell Hemoglobin, PLT: Platelets, WBC: White Blood Cells, LYMP: Lymphocytes, NEUT: Neutrophils, EOS: Eosinophils, BASO: Basophils, MONO: Monocytes, LUC: Large Unstained Cells.

Table S1-2. Hematological parameters after intraperitoneal administration of Cu-NCAB001 in female mice.

Parameters	Day 1 (n = 10)			Day 14 (n = 5)	
	Vehicle	62.5 μg/kg	625 μg/kg	Vehicle	625 μg/kg
RBC 10E4/μL	918 ± 40	936 ± 45	926 ± 53	927 ± 16	937 ± 70
HGB g/dL	14.7 ± 0.5	15.0 ± 0.7	14.7 ± 0.5	14.5 ± 0.4	14.7 ± 0.7
HCT %	47.7 ± 1.8	48.6 ± 1.9	48.2 ± 1.4	47.0 ± 1.0	48.2 ± 2.2
MCV fL	52.0 ± 1.7	52.0 ± 1.2	52.1 ± 1.8	50.7 ± 0.5	51.5 ± 1.6
MCH pg	16.1 ± 0.6	16.1 ± 0.5	16.0 ± 0.5	15.6 ± 0.5	15.8 ± 0.6
Reticulocyte %	3.1 ± 0.6	3.4 ± 0.7	3.2 ± 1.0	3.3 ± 0.3	3.9 ± 1.1
Reticulocyte 10E9/L	288.0 ± 51.5	313.9 ± 66.1	290.2 ± 86.0	307.2 ± 27.6	358.2 ± 81.4

PLT 10E4/μL	114.8 ± 11.9	110.0 ± 14.4	113.4 ± 7.9	102.9 ± 6.5	106.2 ± 10.9
WBC 10E2/μL	36.9 ± 16.9	45.7 ± 17.6	45.8 ± 15.7	39.3 ± 22.1	37.7 ± 9.6
LYMP %	75.8 ± 6.9	73.9 ± 6.2	72.5 ± 6.3	73.1 ± 7.0	74.4 ± 4.3
NEUT %	18.6 ± 7.7	20.5 ± 6.5	22.0 ± 6.5	19.8 ± 7.1	17.6 ± 2.6
EOS %	4.0 ± 1.9	3.7 ± 1.2	3.6 ± 0.8	4.6 ± 2.0	5.7 ± 1.4
BASO %	0.1 ± 0.1	0.1 ± 0.1	0.1 ± 0.1	0.1 ± 0.1	0.1 ± 0.1
MONO %	1.0 ± 0.4	1.2 ± 0.7	1.3 ± 0.7	1.7 ± 0.9	1.8 ± 0.8
LUC %	0.6 ± 0.4	0.6 ± 0.3	0.6 ± 0.3	0.7 ± 0.5	0.4 ± 0.2
LYMP 10E2/μL	28.4 ± 14.3	34.1 ± 13.7	33.4 ± 12.2	28.9 ± 16.2	28.2 ± 7.9
NEUT 10E2/μL	6.4 ± 2.5	9.1 ± 4.0	9.9 ± 4.3	7.6 ± 5.1	6.7 ± 1.8
EOS 10E2/μL	1.5 ± 1.1	1.7 ± 0.7	1.6 ± 0.8	1.9 ± 1.3	2.1 ± 0.6
BASO 10E2/μL	0.0 ± 0.0	0.0 ± 0.1	0.0 ± 0.1	0.0 ± 0.1	0.0 ± 0.0
MONO 10E2/μL	0.3 ± 0.2	0.6 ± 0.4	0.5 ± 0.2	0.6 ± 0.4	0.6 ± 0.3
LUC 10E2/μL	0.2 ± 0.2	0.3 ± 0.2	0.5 ± 0.2	0.2 ± 0.2	0.1 ± 0.1

Values are shown as the mean ± SD. No significant difference in any treated groups from vehicle group. RBC: Red Blood Cells, HGB: Hemoglobin, HCT: Hematocrit, MCV: Mean Red Blood Cell Volume, MCH: Mean Red Blood Cell Hemoglobin, PLT: Platelets, WBC: White Blood Cells, LYMP: Lymphocytes, NEUT: Neutrophils, EOS: Eosinophils, BASO: Basophils, MONO: Monocytes, LUC: Large Unstained Cells.

Table S2-1. Serum biochemical parameters after intraperitoneal administration of Cu-NCAB001 in male mice.

**Parameters**	**Day 1 (n = 10)**			**Day 14 (n = 5)**	
	**Vehicle**	**62.5 μg/kg**	**625 μg/kg**	**Vehicle**	**625 μg/kg**
AST IU/L	38 ± 7	37 ± 6	43 ± 5	46 ± 12	41 ± 3
ALT IU/L	24 ± 4	25 ± 5	27 ± 7	38 ± 14	28 ± 4
LDH IU/L	173 ± 60	186 ± 63	189 ± 48	175 ± 80	155 ± 21
CPK IU/L	49 ± 5	54 ± 17	54 ± 14	44 ± 8	44 ± 9
ALP IU/L	340 ± 78	366 ± 138	358 ± 59	217 ± 15	281 ± 63
T-CHO mg/dL	159 ± 28	168 ± 23	153 ± 9	143 ± 29	132 ± 17
TG mg/dL	65 ± 35	47 ± 22	68 ± 48	72 ± 11	54 ± 18
T-BIL mg/dL	0.1 ± 0.0	0.1 ± 0.0	0.1 ± 0.0	0.1 ± 0.0	0.2 ± 0.1
GUL mg/dL	195 ± 27	205 ± 15	205 ± 15	219 ± 32	200 ± 13
BUN mg/dL	18 ± 2	16 ± 2	17 ± 3	20 ± 4	19 ± 2
CRNN mg/dL	0.10 ± 0.02	0.08 ± 0.01	0.09 ± 0.01	0.13 ± 0.02	0.13 ± 0.02
Na mmol/L	151 ± 1	150 ± 1	150 ± 1	151 ± 1	151 ± 1
K mmol/L	4.7 ± 0.5	4.7 ± 0.4	4.4 ± 0.3	4.6 ± 0.2	4.6 ± 0.3
Cl mmol/L	115 ± 2	114 ± 1	114 ± 2	114 ± 3	114 ± 1
Ca mg/dL	9.7 ± 0.3	9.6 ± 0.2	9.5 ± 0.4	9.1 ± 0.2	9.0 ± 0.3
P mg/dL	8.3 ± 0.9	8.9 ± 0.7	8.3 ± 0.7	7.6 ± 0.7	7.7 ± 0.8
TP g/dL	5.1 ± 0.3	5.1 ± 0.3	5.1 ± 0.2	4.9 ± 0.1	5.0 ± 0.2
ALB g/dL	3.4 ± 0.2	3.4 ± 0.3	3.4 ± 0.2	3.3 ± 0.1	3.4 ± 0.1
A/G	2.0 ± 0.2	2.0 ± 0.2	2.0 ± 0.2	2.0 ± 0.1	2.1 ± 0.2

Values are shown as the mean±SD. No significant difference in any treated groups from vehicle group. AST: Aspartic Aminotransferase, ALT: Alanine Aminotransferase, LDH: Lactate Dehydrogenase, CPK: Creatine Phosphokinase, ALP: Alkaline Phosphatase, T-CHO: Total Cholesterol, TG: Triglyceride, T-BIL: Total Bilirubin, GUL: Glucose, BUN: Blood Urea Nitrogen, CRNN: Creatinine, TP: Total Protein, ALB: Albumin, A/G: Albumin/Globulin ratio.

Table S2-2. Serum biochemical parameters after intraperitoneal administration of Cu-NCAB001 in female mice.

Parameters	Day 1 (n=10)			Day 14 (n=5)	
	Vehicle	62.5 μg/kg	625 μg/kg	Vehicle	625 μg/kg
AST IU/L	46 ± 6	48 ± 9	50 ± 13	48 ± 4	47 ± 3
ALT IU/L	23 ± 5	22 ± 4	28 ± 12	27 ± 3	26 ± 2
LDH IU/L	185 ± 70	169 ± 43	210 ± 82	136 ± 31	215 ± 63 †
CPK IU/L	368 ± 96	395 ± 75	392 ± 151	47 ± 8	55 ± 9
ALP IU/L	368 ± 96	395 ± 75	392 ± 151	344 ± 119	333 ± 20
T-CHO mg/dL	106 ± 22	106 ± 16	106 ± 20	87 ± 9	119 ± 33
TG mg/dL	42 ± 24	46 ± 15	48 ± 25	64 ± 9	66 ± 14
T-BIL mg/dL	0.1 ± 0.0	0.1 ± 0.0	0.1 ± 0.0	0.1 ± 0.0	0.1 ± 0.0
GUL mg/dL	191 ± 8	196 ± 26	197 ± 22	191 ± 22	199 ± 16
BUN mg/dL	18 ± 3	18 ± 4	18 ± 2	19 ± 3	15 ± 3
CRNN mg/dL	0.11 ± 0.02	0.11 ± 0.01	0.10 ± 0.01	0.15 ± 0.03	0.15 ± 0.02
Na mmol/L	150 ± 2	150 ± 2	150 ± 1	151 ± 1	150 ± 1
K mmol/L	4.2 ± 0.4	4.3 ± 0.6	4.4 ± 0.2	4.1 ± 0.4	3.9 ± 0.3
Cl mmol/L	116 ± 2	116 ± 2	115 ± 2	115 ± 1	115 ± 2
Ca mg/dL	9.6 ± 0.3	9.4 ± 0.2	9.6 ± 0.2	9.0 ± 0.1	8.9 ± 0.1
P mg/dL	7.9 ± 0.9	7.9 ± 0.9	8.4 ± 1.0	6.8 ± 0.2	7.3 ± 0.6
TP g/dL	5.1 ± 0.3	4.9 ± 0.2	4.9 ± 0.2	4.9 ± 0.2	5.0 ± 0.2
ALB g/dL	3.6 ± 0.2	3.5 ± 0.1	3.5 ± 0.1	3.6 ± 0.1	3.6 ± 0.2
A/G	2.5 ± 0.3	2.6 ± 0.3	2.6 ± 0.2	2.7 ± 0.2	2.6 ± 0.3

Values are shown as the mean ± SD. † $p \leq 0.05$ vs. vehicle group (t test, two-side). AST: Aspartic Aminotransferase, ALT: Alanine Aminotransferase, LDH: Lactate Dehydrogenase, CPK: Creatine Phosphokinase, ALP: Alkaline Phosphatase, T-CHO: Total Cholesterol, TG: Triglyceride, T-BIL: Total Bilirubin, GUL: Glucose, BUN: Blood Urea Nitrogen, CRNN: Creatinine, TP: Total Protein, ALB: Albumin, A/G: Albumin/Globulin ratio.

Table S3-1. Organ weights after intraperitoneal administration of Cu-NCAB001 in male mice.

Parameters	Day 1 (n=10)			Day 14 (n=5)	
	Vehicle	62.5 μg/kg	625 μg/kg	Vehicle	625 μg/kg
Brain mg	475 ± 19	484 ± 24	479 ± 25	476 ± 18	489 ± 9
Thymus mg	50 ± 17	58 ± 9	55 ± 10	48 ± 8	54 ± 7
Heart mg	142 ± 11	143 ± 10	138 ± 9	153 ± 23	159 ± 10
Lung mg	180 ± 16	178 ± 9	185 ± 31	198 ± 22	193 ± 23
Liver g	1.49 ± 0.15	1.59 ± 0.12	1.51 ± 0.13	1.78 ± 0.21	1.68 ± 0.17
Spleen mg	78 ± 11	83 ± 13	84 ± 16	92 ± 26	96 ± 23
Kidney-RL mg	453 ± 49	462 ± 27	455 ± 38	507 ± 29	510 ± 47
Testis-RL mg	201 ± 49	200 ± 26	202 ± 30	243 ± 42	243 ± 30

Values are shown as the mean ± SD. No significant difference in any treated groups from vehicle group. RL: Right and Left.

Table S3-2. Organ weights after intraperitoneal administration of Cu-NCAB001 in female mice.

Parameters	Day 1 (n=10)			Day 14 (n=5)	
	Vehicle	62.5 μg/kg	625 μg/kg	Vehicle	625 μg/kg
Brain mg	470 ± 21	476 ± 26	469 ± 13	485 ± 18	481 ± 23
Thymus mg	57 ± 17	53 ± 14	61 ± 12	55 ± 9	49 ± 9
Heart mg	116 ± 12	118 ± 6	121 ± 12	140 ± 14	131 ± 15
Lung mg	159 ± 13	160 ± 18	157 ± 14	170 ± 12	169 ± 10
Liver g	1.14 ± 0.14	1.13 ± 0.10	1.20 ± 0.16	1.32 ± 0.07	1.39 ± 0.21
Spleen mg	75 ± 13	85 ± 15	88 ± 21	93 ± 16	100 ± 13
Kidney-RL mg	293 ± 30	300 ± 24	322 ± 22 †	333 ± 26	334 ± 42

Values are shown as the mean ± SD. † $p \leq 0.05$ vs. vehicle group (Dunnett test, two-side). RL: Right and Left.
